# Stereotactic body radiation therapy in the treatment of ovarian cancer

**DOI:** 10.1186/s13014-020-01564-w

**Published:** 2020-05-13

**Authors:** Roman O. Kowalchuk, Michael R. Waters, K. Martin Richardson, Kelly Spencer, James M. Larner, William P. Irvin, Charles R. Kersh

**Affiliations:** 1 University of Virginia / Riverside, Radiosurgery Center, Newport News, VA USA; 2 Department of Radiation Oncology, University of Virginia, Charlottesville, VA USA; 3 Department of Gynecologic Oncology, Riverside Regional Medical Center, Newport News, USA

**Keywords:** SBRT, SABR, Ovarian cancer, Radiation oncology

## Abstract

**Background:**

This study evaluates the outcomes and toxicity of stereotactic body radiation therapy (SBRT) in ovarian cancer.

**Methods:**

This retrospective analysis considered all patients treated with SBRT from 2009 to 2018 with a primary ovarian tumor. Follow-up included PET-CT and CT scans at 2–3 month intervals. Statistical analysis primarily consisted of univariate analysis, Cox proportional hazards analysis, and the Kaplan-Meier method.

**Results:**

The study included 35 patients with 98 treatments for lymph nodes (51), local recurrence (21), and de novo solid metastases (26). Median biologically effective dose (BED), gross tumor volume, and planning target volume were 38.40 Gy, 10.41 cc, and 25.21 cc, respectively. 52 lesions showed complete radiographic response, and two-year local control was 80%. Median overall survival (OS) was 35.2 months, and two-year progression-free survival (PFS) was 12%. On univariate analysis, Eastern Cooperative Oncology Group performance status > 0 was predictive of decreased OS (*p* = 0.0024) and PFS (*p* = 0.044). Factors predictive of local failure included lower BED (*p* = 0.016), treatment for recurrence (*p* = 0.029), and higher pre-treatment SUV (*p* = 0.026). Kaplan-Meier analysis showed BED ≤35 Gy (*p* < 0.005) and treatment for recurrence (*p* = 0.01) to be predictive of local failure. On Cox proportional hazards analysis, treatment of lymph nodes was predictive of complete radiographic response (hazard ratio (HR) = 4.95), as was higher BED (HR = 1.03). Toxicity included 27 cases of grade < 3 toxicity, and one grade 5 late toxicity of GI bleed from a radiation therapy-induced duodenal ulcer.

**Conclusions:**

SBRT provides durable local control with minimal toxicity in ovarian cancer, especially with BED > 35 Gy and treatment for lymph nodes.

## Introduction

Ovarian cancer is a heterogeneous disease without clear screening guidelines for early detection [1–5]. It is often diagnosed at advanced stages of disease, resulting in relapse in 75% of patients and the frequent need for salvage therapy [6–9]. The biology of relapse may involve treatment resistance stemming from a sub-population of cancer stem cells or changes within the tumor microenvironment and extracellular matrix [8–11]. Advances in molecularly targeted treatments, however, have helped extend patient survival [12–14].

A variety of radiation techniques are currently being considered in the management of ovarian cancer, including intensity-modulated radiotherapy, stereotactic body radiotherapy (SBRT), and low-dose hyperfractionation combined with targeted agents [15, 16]. Postoperative pelvic radiotherapy and radiotherapy after chemotherapy in advanced disease have also emerged as possible uses for radiotherapy [17]. In one study, involved-field radiation therapy for locoregionally-recurrent ovarian cancer demonstrated an impressive 5 year in-field disease control rate of 71%, and another analysis showed a 65% response rate to radiation therapy for recurrent epithelial ovarian cancer [18, 19]. Unfortunately, stringent guidelines regarding the use of radiotherapy in ovarian cancer do not exist. Therefore, available studies investigating the use of radiation in ovarian cancer include a range of radiotherapy techniques and a mix of ovarian cancer subtypes [20–22].

SBRT, also known as stereotactic ablative radiotherapy (SABR), is a radiation therapy strategy that involves delivering high doses of radiation to the tumor in relatively few treatments (about 3–6) [23]. SBRT has shown efficacy in the treatment of oligometastases from colorectal cancer, especially when the target of treatment has been a lymph node or small tumor [24, 25]. Further studies have included other primary solid tumors, including the lung and prostate. SBRT may be most efficacious in carefully selected patients with a disease-free interval of ≥12 months, control of the primary tumor, small lesions, a limited number of lesions, and a higher delivered radiation dose [26–30]. The SABR-COMET trial, a randomized phase 2 study, demonstrated that SABR in patients with a limited number of metastatic lesions resulted in improved overall survival [31].

The role of SBRT in metastatic gynecologic cancer has also been studied and found to be safe and efficacious, particularly when targeting lymph node recurrences [32, 33]. The largest analysis considering SBRT for ovarian cancer metastases was a multi-institutional study that reported completed response in 65.2% of treatments. Lymph node disease was correlated with complete response, but further single institutional study is needed [34–37]. This study aims to analyze the outcomes and toxicity of SBRT in ovarian cancer at a single high-volume stereotactic radiosurgery center and discern factors predictive of overall survival (OS), local control, and radiographic response.

## Methods

This retrospective analysis considered all patients at a single institution treated with SBRT for a primary ovarian tumor from April 2009–November 2018. An intention-to-treat model was undertaken, so each patient treated was included in the analysis. No exclusion criteria were specified, so patients were included regardless of the SBRT target (lymph node, recurrence, or de novo solid metastasis). Treatment for recurrence was further delineated into local, regional, and distant recurrence. Local recurrence was defined as a previously treated lesion that had grown in size on imaging. Previous treatment may have included surgical resection, chemotherapy, and radiation therapy. New lesions arising within the planning target volume (PTV) of previous radiotherapy were also included as local recurrences. Regional recurrence was denoted as new disease in proximity to a previous lesion and without an anatomic barrier separating the lesions. For instance, a new abdominal lesion in the setting of prior abdominal disease would be deemed a regional recurrence. Other disease progression not meeting these criteria was denoted as distant recurrence. Patients of all pathological subtypes were included, and the corresponding histology was recorded.

The number of treated lesions and the performance status were recorded for each patient, and no restrictions were made regarding the maximum lesion size for inclusion in the study. The authors decided that since no explicit exclusion criteria regarding lesion size have been developed in the setting of ovarian cancer, all patients treated with SBRT for ovarian cancer should be included. Pre- and post-chemotherapy CA125 levels were noted, as was each patient’s response to chemotherapy. Prior and subsequent treatments were also included, as were each patient’s platelet, lymphocyte, and neutrophil counts prior to and after treatments (when available). Follow-up included PET-CT and CT imaging at 2–3 month intervals, and standardized uptake values (SUV) and CA125 lab values were recorded prior to and after treatment. PET-CT generally involved total body scans using ^18^FDG, and the target of the CT scan varied, depending on the location of the SBRT target. Most frequently, CT abdomen/pelvis was used. Key dosimetric data included the dose, fractions, days between fractions, and the dose-fractionation scheme of any prior radiation. An alpha/beta ratio of 10 Gy was used for calculations of the biologically effective dose (BED), using the formula BED = total dose * (1 + dose per fraction / alpha/beta ratio). The study was exempt by the institutional review board.

Patients were immobilized prior to planning the CT scan using a full-body vacuum bag system for position stabilization and consistency. Dose was prescribed to the PTV, which was defined as the gross tumor volume (GTV) plus 3–5 mm of margin to account for uncertainties in imaging and localization. In general, a three-dimensional conformal treatment planning approach was used with non-coplanar gantry angles to minimize dosimetric overlap of entrance and exit portals. Intensity-modulated radiotherapy and volumetric-modulated arc therapy were considered, but they were not used in this setting. Treatments were instead completed with multiple non-coplanar static gantry delivery. SBRT was delivered using a 6MV photon beam on a linear accelerator with a 2.5 mm–4 mm width multi-leaf collimator for custom shaping of portals. An on-board cone-beam CT was used prior to treatment to align the patient. It was also used several times (generally 2–4) during treatment to correct for intra-fraction movement. A robotic couch with six degrees of freedom assisted in the alignment of the patient and localization of the target to the planning CT. Treatments were generally delivered once weekly. The median days between fractions was reported in a non-inclusive manner, such that there were 6 days between once-weekly treatments.

The key endpoints of the analysis involved overall survival (OS), local control, and radiographic response. Progression-free survival (PFS) and time to salvage were secondary endpoints. Two-year local control and two-year PFS were determined using the Kaplan-Meier method. Patient follow-up was generally conducted by radiation oncology, hematology-oncology, or gynecologic-oncology. Imaging was used to classify treatment response as progressive, stable, partial, or complete. Any treatment failure was also noted on imaging, and it was distinguished as local, regional, or distant failure. Acute and chronic toxicity findings were tabulated according to the Common Terminology Criteria for Adverse Events v5.0. Ninety days was the cutoff for delineating acute from chronic toxicity.

Statistical analysis was conducted for each treated lesion to analyze local control and radiographic response, and survival analysis was performed for each patient treated. Predictive factors for overall survival and local control were assessed via Cox proportional hazards regression analysis and univariate analysis, with an alpha value of 0.05. Local control and overall survival were further described using the Kaplan-Meier method, including distinctions between the overall survival and local control of different subgroups of the cohort.

## Results

Thirty-five patients with ninety-eight treated lesions were included. Seventeen patients were treated for one lesion, but eighteen patients were treated for at least two distinct lesions with SBRT, including seven patients receiving at least 5 treatments. Of patients with multiple treatments, five patients had only synchronous lesions, seven had only metachronous lesions, and six suffered from both synchronous and metachronous lesions. The time of presentation for SBRT was a median 44.81 months after primary diagnosis. Only two patients presented for SBRT within 12 months of primary diagnosis. Twenty-one (60%) patients had Eastern Cooperative Oncology Group (ECOG) performance status 0 at the time of consultation for SBRT. Five patients (14%) had a diagnosed BRCA1 mutation, and 2 (6%) were positive for homologous recombination deficiency. The majority of patients (66%) had a serous papillary adenocarcinoma on pathologic review, and the other subtypes included clear cell, transitional cell, granulosa cell, endometrioid, carcinosarcoma, mucinous, and other poorly differentiated tumors (Table 1).

**Table 1:** Patient demographics are carefully explored

Patient Demographics	Number	Rate
Total patients	35	
Total lesions	98	
Patients with 1 lesion	17	49%
Patients with 2 lesions	6	17%
Patients with 3 lesions	2	6%
Patients with 4 lesions	3	9%
Patients with ≥5 lesions	7	20%
Median age at diagnosis (years)	62.8 (32.7–80.6)	
ECOG 0	21	60%
ECOG 1	11	31%
ECOG 2	1	3%
ECOG 3	2	6%
ECOG 4	0	0%
**Histology**		
Serous papillary adenocarcinoma	23	66%
Mixed	1	3%
Clear cell	1	3%
Transitional cell	1	3%
Granulosa cell	1	3%
Endometrioid	2	6%
Carcinosarcoma (mixed Mullerian)	2	6%
Poorly differentiated / Undifferentiated	3	9%
Mucinous	1	3%
Positive BRCA 1	5	14%
Positive HRD	2	6%
Neoadjuvant chemotherapy	8	23%
No neoadjuvant chemotherapy	27	77%
Gross residual post-op	14	40%
No gross residual post-op	21	60%
Median post-chemotherapy CA125	18	
Median pre-SBRT SUV	5.5	
**Staging at Diagnosis**		
Stage I	5	14%
Stage II	3	9%
Stage III	18	51%
Stage IV	9	26%
Grade 1	1	3%
Grade 2	4	11%
Grade 3	26	74%
Grade NA	4	11%

Pre-SBRT chemotherapy and further tumor details were also reported for each patient. At diagnosis, most patients presented at advanced stages of disease (51% at stage 3 and 26% at stage 4). Of these, twenty-two treated lesions initially presented with nodal spread, and only ten presented with T stage less than 3. Most tumors were grade 3 on pathologic review (74%), and only one case involved a well-differentiated, grade 1 tumor. Surgical interventions were varied. They included a range of primary goals: exploration (11%), debulking (34%), lymph node resection or sampling (14%), omentectomy (31%), peritoneal stripping (11%), and bowel resection (37%). Some form of total abdominal hysterectomy with or without unilateral or bilateral oophorectomy was also undertaken in 49% of cases. Many patients (40%) still had gross residual disease after the initial debulking surgery, but 78.4% of patients were sensitive to chemotherapy. All but one chemotherapy regimen included carboplatin. The treatments included carboplatin and paclitaxel (83%), carboplatin alone (6%), carboplatin and docetaxel (9%), carboplatin and gemcitabine (3%), cisplatin and paclitaxel (3%), and carboplatin, gemcitabine, and bevacizumab (3%). Four patients used two primary chemotherapy regimens, and the median number of cycles of chemotherapy was six. CA125 was reduced after chemotherapy in the majority of cases. Six patients (17%) had received radiotherapy prior to SBRT. Five of these treatments were related to ovarian cancer, including whole pelvis radiotherapy, radiotherapy for bone metastases, and brachytherapy.

The median dose and fractionation were 24 Gy in 4 fractions, with a median 4 days between fractions. The most common dose-fractionation schemes were 20–24 Gy in 3–4 fractions (55%), 30–40 Gy in 3–5 fractions (26%), and 15–18 Gy in 3–4 fractions (14%). Median BED was 38.40 Gy, and median GTV and PTV were 10.41 and 25.21 cc, respectively. There was no limit imposed on the maximum GTV for inclusion in the study, so large tumors were also included (maximum GTV of 272.37 cc). Even so, lesions were generally smaller. Only seven treatments involved a GTV > 50 cc. Concurrent chemotherapy was involved in only six cases. Treatments were for lymph nodes (51), local recurrence (21), and de novo solid metastases (26). Treatments for local recurrence generally involved abdominal soft tissue (62%) or perigastric (19%) targets after surgical intervention and chemotherapy. In six instances, local recurrence involved re-treatment with SBRT, and in fifteen cases, radiotherapy followed treatment failure of surgery or chemotherapy. Of patients treated for de novo metastases, 77% of treatments targeted liver metastases, with the spleen, lung, and bone comprising the other treated locations (Table 2). Though there were no differences in GTV or PTV between treatments for recurrence and de novo solid metastases, treatments for recurrence had increased GTV (*p* = 0.03) and PTV (*p* = 0.003) relative to treatments for lymph nodes.

**Table 2:** The details of the radiation therapy are tabulated

Dosimetric Characteristics	Number	Rate
Median dose (Gy)	24 (12–40)	
Median fractions	4 (3–6)	
Median BED (Gy)	38.40 (16.80–84.38)	
Median GTV (cc)	10.41 (0.30–272.37)	
Median PTV (cc)	25.21 (1.79–393.07)	
Mean days between fractions	5.39	
Concurrent chemotherapy	6	6%
**SBRT Target**		
Extrapelvic	75	77%
Intrapelvic	23	23%
Local recurrence	21	21%
Lymph node	51	52%
Spleen	2	2%
Liver	20	20%
Lung	2	2%
Bone	2	2%

**Fig. 1:**
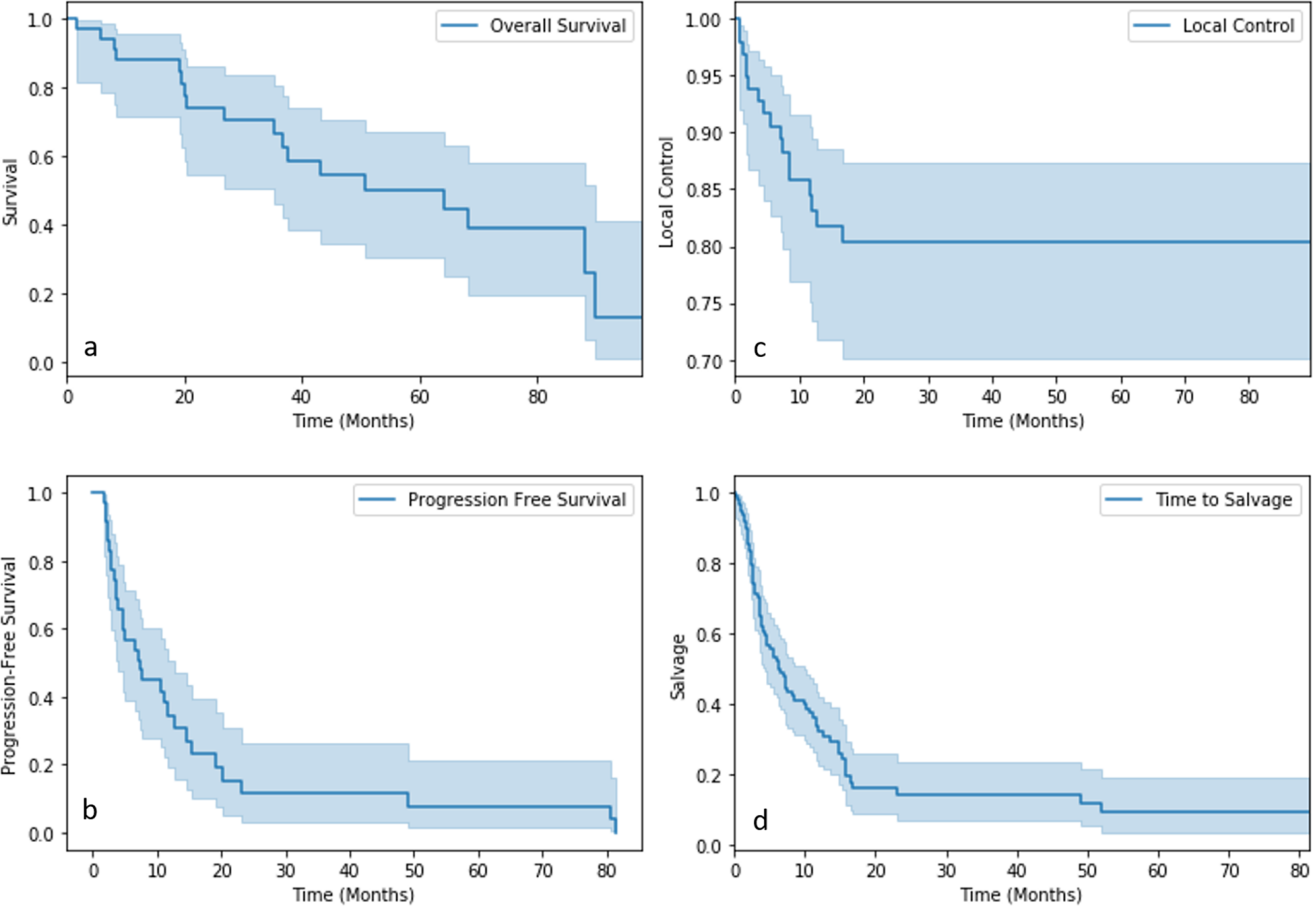
Kaplan-Meier curves of OS, PFS, local control, and time to salvage are shown

Median follow-up was 33.67 months. Median OS was 35.2 months, and two-year PFS was 12%. OS at 2 years was 60%. On imaging follow-up, 52 lesions (53%) showed complete radiographic response, and partial response, lesion stability, and tumor progression were reported in 21, 12, and 1% of cases, respectively. Lesion size decreased a median 29.2% after SBRT on the first imaging follow-up. Local control was maintained in 81 cases, resulting in a two-year local control rate of 80% (Table 3). These results were demonstrated using the Kaplan-Meier method (Fig. 1). Failure patterns included distant (39%), regional (32%) and local (17%) relapse. Subsequent treatments included salvage chemotherapy (63%), radiotherapy (36%), and surgery (6%). The median time to salvage therapy was 4.29 months. Neutrophil, platelet, and lymphocyte counts were also recorded prior to and after SBRT. These counts decreased by a median 10.53, 8.57, and 14.29%.

**Table 3:** Patient outcomes, including imaging response, disease relapse, and survival are considered

Outcome	Number	Rate
No relapse	18	18%
Relapse	80	82%
Two-year local control		80%
Local relapse	17	17%
Regional relapse	31	32%
Distant relapse	38	39%
Salvage chemotherapy	62	63%
Salvage radiotherapy	35	36%
Salvage surgery	6	6%
No salvage treatment	9	9%
Median time to salvage (months)	4.29 (0.26–51.98)	
Alive	18	51%
Deceased	17	49%
Median overall survival (months)	35.19 (1.81–97.64)	
Two-year PFS		12%
**Imaging Response**		
Progressive	1	1%
Stable	12	12%
Partial	21	21%
Complete	52	53%
No imaging follow-up	12	12%
Initial median target size (cm)	2.13 (0.40–15.10)	
Target size after SBRT (cm)	1.50 (0–7.37)	
Reduction in target size (%)	29.17	
Median neutrophil count reduction	10.53%	
Median platelet count reduction	8.57%	
Median lymphocyte count reduction	14.29%

**Fig. 2:**
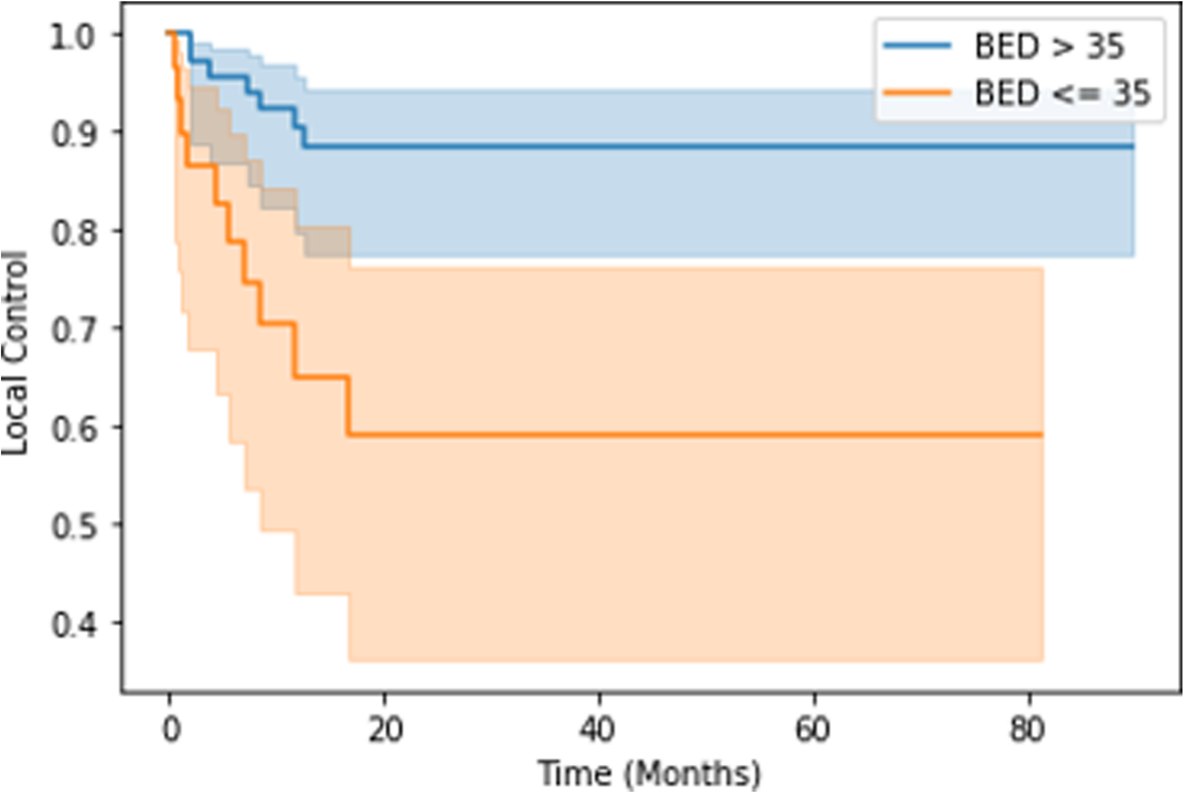
Kaplan-Meier analysis demonstrates improved local control with BED > 35 Gy (*p* < 0.005)

**Fig. 3:**
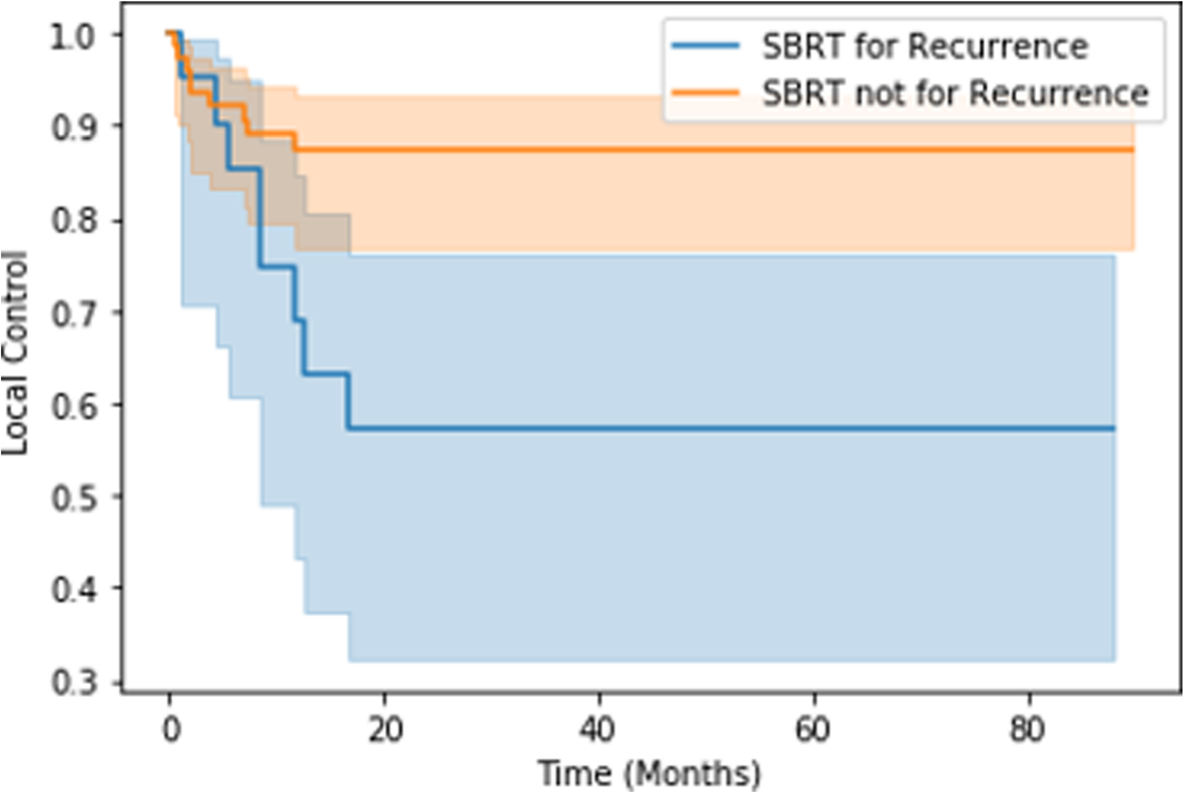
Kaplan-Meier analysis shows decreased local control with SBRT for recurrence (*p* = 0.01)

Univariate analyses were conducted, in addition to Cox proportional hazards regression analysis. ECOG > 0 was the only factor predictive of decreased OS (*p* = 0.0024) and PFS (*p* = 0.044). On the other hand, factors predictive of local failure on univariate analysis included lower BED (*p* = 0.016), treatment for recurrence (*p* = 0.029), and higher pre-treatment SUV (*p* = 0.026). Treatment of liver metastases was also predictive of local control (*p* = 0.025). On Cox proportional hazards analysis, larger GTV volume was predictive of local failure (hazard ratio (HR) =1.04), but the prognostic impact of the treatment target led to wide confidence intervals (Table 4). On Kaplan-Meier analysis, BED ≤35 Gy was predictive of local failure (*p* < 0.005), as was treatment for recurrence (*p* = 0.01) (Figs. 2 and 3). Kaplan-Meier analysis failed to demonstrate any difference in local control rates between patients with longer versus shorter times to treatment or any of the other factors previously discussed.

**Table 4:** The results of the univariate and Cox proportional hazards analyses are shown. Bolded *p* values indicate statistical significance

	Local failure		Complete radiographic response
Predictive factor	Univariate analysis (*p* value)	Hazard ratio (confidence interval)	Univariate analysis (*p* value)
BED (Gy)	**0.016**	0.95 (0.91–1.00)	0.52
- BED ≤35	**0.017**		0.087
GTV	0.22	1.04 (1.00–1.08)	0.19
PTV	0.62	0.98 (0.95–1.01)	0.49
Target size	0.16	1.04 (0.82–1.31)	0.071
Treatment for recurrence	**0.029**	0.71 (0.07–7.79)	**0.003**
Treatment for lymph node	0.34	0.36 (0.04–3.32)	**0.027**
Treatment for liver metastasis	**0.025**	0.20 (0.01–3.65)	0.79
Time from primary diagnosis	0.20	1.00 (0.99–1.02)	0.49
Higher pre-treatment SUV	**0.026**		**0.035**

**Fig. 4:**
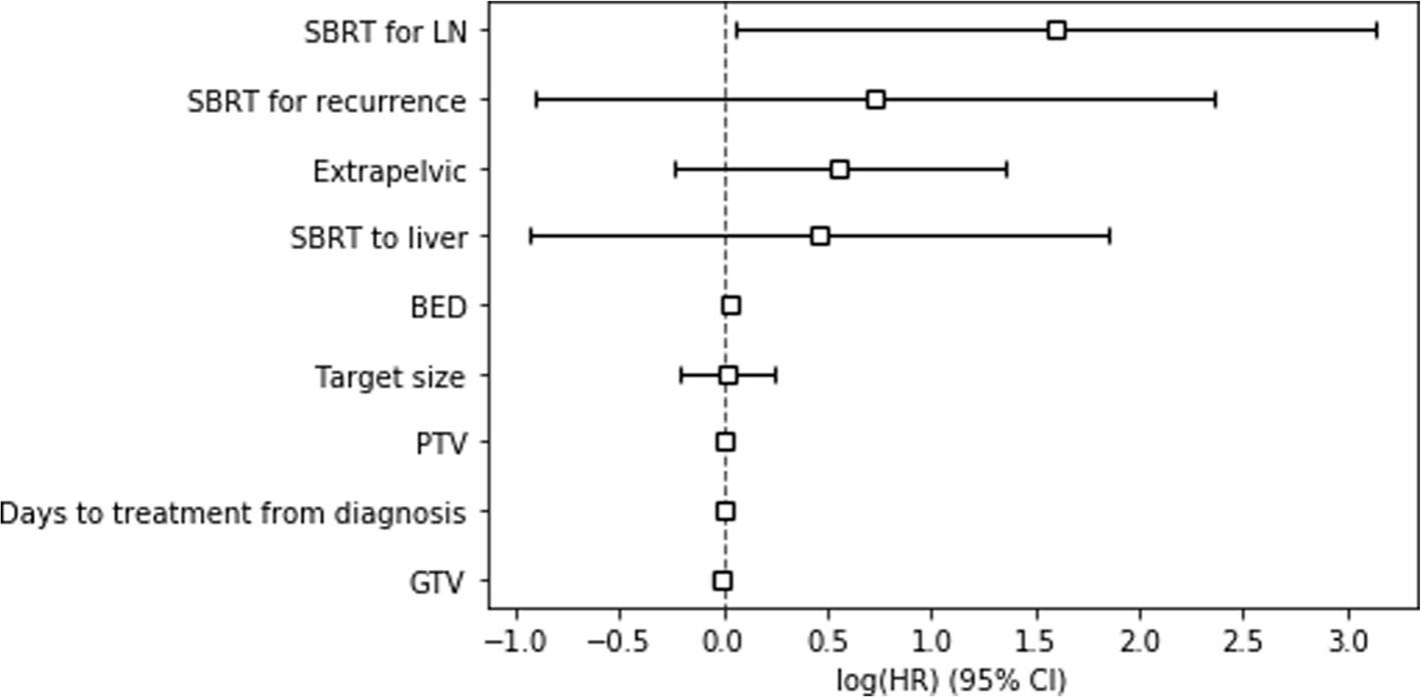
Cox proportional hazards analysis for predictive factors of complete radiographic response is displayed

Factors predictive of incomplete radiographic response included treatment for recurrence (*p* = 0.0030) and higher pre-treatment SUV (*p* = 0.035). Additionally, target size trended towards significance (*p* = 0.071). On Cox proportional hazards analysis, treatment of lymph nodes was predictive of complete radiographic response (HR = 4.95), as was higher BED (HR = 1.03) (Fig. 4). In the hazards analysis, SBRT for a liver metastasis target was used as a surrogate for SBRT for metastasis because the analysis using SBRT for metastases in this context demonstrated high collinearity, and 20 of 26 cases (77%) of treatment for metastasis involved treatment of a liver mass. Additionally, pre-treatment SUV was not included in the hazards analysis because not all patients had a pre-treatment PET scan. No differences in outcomes were seen depending on the changes in neutrophil, platelet, or lymphocyte counts.

Toxicity included 22 cases of acute grade 1 toxicity, five instances of acute grade 2 toxicity, and one late grade 5 late toxicity of GI bleed from what was believed to be a radiation therapy-induced duodenal ulcer. This case involved the delivery of 18 Gy in three fractions to a 1.93 cm target in the porta hepatis of the liver. The patient had received a separate SBRT treatment one month prior to a distinct porta hepatis lesion. This lesion had been treated with 21 Gy in three fractions to a PTV of 58.24 cc. The most common acute grade 1 toxicities were seven instances of nausea and vomiting and seven of fatigue. Other acute grade 1 side effects included pain (4 cases), erythema (2 cases), and diarrhea (2 cases). Acute grade 2 toxicity involved nausea and vomiting (three cases) and pain (two cases) (Appendix).

## Discussion

Local failure was one of the principal endpoints of this study, and the results were encouraging, with 83% local control at a median follow-up time of 33.67 months. This follow-up time is quite extensive, as many studies regarding SBRT for ovarian cancer include only 12–24 months of follow-up. The largest study to date of SBRT in oligometastatic ovarian cancer was written by Macchia, et al., a multi-institutional study of 15 centers considering 446 lesions treated in 261 patients, and the results were quite similar to those presented here. At a median follow-up of 22 months, the 24-month actuarial local control rate was 81.9%. Local control was associated with complete radiographic response and total dose > 25 Gy [34]. Iftode et al. reported a local control rate of 92.9% at 1 year, and Trippa et al. found a local control of 73% in a subset of lymph node disease secondary to ovarian cancer treated with SBRT [35, 36]. Trippa et al. also determined that higher dose was correlated with improved local control. Finally, Lazzari et al. presented the largest single-institutional study to date involving SBRT in oligometastatic ovarian cancer, but they only reported a complete radiographic response in 60% at 17.4 months of follow-up [34]. Overall, the local control rate from this study is consistent with the literature in that SBRT offers treatment efficacy in metastatic ovarian cancer. Nonetheless, there are very few studies evaluating the outcomes of SBRT in this context. For this reason, it is unsurprising that there is heterogeneity between the inclusion and exclusion criteria in the studies discussed above. Trippa et al. only presented data regarding the treatment of lymph node disease, and the evaluation of a study considering results from 15 institutions is also complicated by the differences in treatment planning between those centers [34, 37]. Other papers have considered metastases from a variety of primary gynecologic cancers [36].

In this analysis, factors predictive of local failure included BED ≤35 Gy, larger GTV, treatment for recurrence, and higher pre-treatment SUV. Treatment of liver metastases was also predictive of local control. Unfortunately, it is difficult to directly compare these results to literature values because the few studies concerning this topic have either not included sufficient numbers of treatments for such analyses or have focused on a different endpoint. Radiographic response has been used as a surrogate for local control, and it was the chief endpoint presented by Lazzari et al. Macchia et al. found that age ≤ 60 years, PTV ≤ 18 cc, lymph node disease, and BED > 70 Gy were associated with complete radiographic response. These factors were not found to be directly correlated with local control. In this analysis, complete response was observed in 52 cases, which is 53% of all cases included in the study but 60% of all cases with follow-up imaging. This result is quite consistent with literature values of 61.8–65.2% [34, 35]. Here, factors predictive of incomplete radiographic response included treatment for recurrence and higher pre-treatment SUV. The increased local failure with higher pre-treatment SUV points towards a potential prognostic role for PET imaging. Lung lesion shrinkage of at least 20% at the last session of SABR on cone-beam CT has been investigated as a predictive factor of complete response [38]. Additionally, the maximum and mean SUV values on ^18^FDG-PET/CT have also been shown to be predictive of complete response [39]. These findings point towards the need for further investigation of pre-treatment SUV as a prognostic factor.

The concept of higher dose delivery resulting in improved local control and even improved survival rates has been observed with SBRT in other solid malignancies [31, 37, 40]. For instances, high rates of treatment efficacy have been observed for liver metastases with BED > 100 Gy [41]. Smaller tumor size or treatment volume intuitively may relate to improved treatment efficacy for a variety of reasons [25, 37]. For instance, higher dose may be more feasibly delivered to this location while minimizing toxicity. Finally, the result that SBRT is more effective for lymph node disease and less efficacious when treatment is for cancer recurrence is intriguing. Results for SBRT treatment of lymph node disease from a variety of primary cancers have been encouraging, so it is not surprising that results in this patient subgroup were positive [24, 28, 33]. Salvage treatments have been somewhat more complex, as they present a different set of challenges. These include difficulties with delivering high dose radiation again within a prior radiation treatment field and concerns about the radioresistance of the tumor. This patient subgroup of ovarian cancer patients who need treatment of recurrent disease requires further study because their outcomes may be worse than those receiving primary treatment with SBRT, and relapse rates within 2 years of primary therapy are about 75% [7]. One potential contributing factor to this difference could be the treatment volume because treatments for recurrence had increased GTV and PTV relative to treatments for lymph nodes. Even so, treatment for recurrence was independently predictive of local failure on Kaplan-Meier analysis, and treatment for lymph nodes predicted for complete radiographic response. Volume did not have a statistically significant impact on complete radiographic response or local control via the Kaplan-Meier method, so it is possible that the treatment target is the dominant factor. Further study into this distinction is recommended.

Survival in this patient cohort was quite impressive, with a median overall survival of 35.2 months. This value is much higher than the poor survival generally presented in the literature [3, 9]. ECOG > 0 was the only factor predictive of decreased OS (*p* = 0.0024) and PFS (*p* = 0.044). It is possible that the patients in this study had relatively high performance statuses compared with other ovarian cancer patients, as 60% of patients had ECOG 0 and 31% had ECOG 1. On the other hand, the majority of patients in this study (51%) were treated with SBRT for multiple lesions. This was in the context of a progression-free survival of 7.2 months and median time to salvage of 4.29 months. Lazzari et al. noted a similar treatment-free interval after SBRT of 7.4 months [35]. Large database study will likely play a key role in further studying overall survival [42]. The development of a nomogram assessing the role of pretreatment characteristics indicated that performance status, ascites, size of the largest tumor, CA125, platinum-free interval, and primary platinum resistance were the significant predictors for OS [43]. Further study of these factors may help optimize patient selection of ovarian cancer patients for SBRT.

Toxicity in this study was quite low overall, with 22 cases of grade 1 toxicity, five instances of grade 2 toxicity, and one grade 5 late toxicity of GI bleed from what was believed to be a radiation therapy-induced duodenal ulcer. The most common side effects of nausea, vomiting, and fatigue were not severe. These were also relatively non-specific symptoms that may have been related to the primary disease process. There was only 1 case of grade ≥ 3 toxicity, which involved the grade 5 GI bleed. Similarly, Macchia et al. reported that only 20.7% of patients suffered from any toxicity, none of which was grade ≥ 3 [34].​ Lazzari et al. also noted no grade ≥ 3 toxicity for SBRT in oligometastatic ovarian cancer [35]. These findings together point towards the safety of this technique.

This study suffers from some limitations regarding the inclusion of only 35 distinct patients. This could serve to depress the ability of the authors to determine prognostic factors regarding survival and could lead to high variance in the survival outcomes. Additionally, the inclusion of treatments involving local relapse may represent a significance weakness of the present analysis. It was shown that treatments for recurrence had increased rates of local failure, and they may represent a distinct clinical question from treatments for lymph nodes or de novo solid metastases. Further study is recommended to clarify this distinction, as well as the role of systemic treatment in this setting. Relatively low prescription doses were used in this study. It is possible that higher doses might have improved the complete response rate, and further study into the use of higher doses is recommended.

## Conclusions

The authors were able to analyze a large cohort of lesions treated with SBRT from primary ovarian cancer at a single institution and present the findings that the technique was associated with high rates of local control, impressive survival, and minimal toxicity. Patients with BED ≤35 Gy, larger GTV, and treatment for recurrence had increased local failure while those with treatments for lymph node disease had improved local control.

## Data Availability

The datasets generated during and/or analyzed during the current study are available from the corresponding author on reasonable request.

## Appendix

**Table 5:** Toxicity results are demonstrated

Acute	Grade 1	Grade 2	Grade 3–4	Grade 5
Nausea/Vomiting	7	3	0	0
Fatigue	7	0	0	0
Pain	4	2	0	0
Erythema	2	0	0	0
Diarrhea	2	0	0	0
Total	22	5	0	0
**Late**				
Duodenal ulcer	0	0	0	1
Total	0	0	0	1

## Abbreviations

SBRT: Stereotactic body radiation therapy
PET: Positron emission tomography
CT: Computed tomography
BED: Biologically effective dose
GTV: Gross tumor volume
PTV: Planning target volume
OS: Overall survival
PFS: Progression-free survival
SUV: Standardized uptake values
CA125: Cancer antigen 125
SABR: Stereotactic ablative radiotherapy
ECOG: Eastern Cooperative Oncology Group
